# Returning to Sport After Anterior Cruciate Ligament Reconstruction in Physically Active Individuals

**DOI:** 10.7759/cureus.10466

**Published:** 2020-09-15

**Authors:** Muath M Alswat, Osama Khojah, Anas M Alswat, Abdulrhman Alghamdi, Mohab S Almadani, Ammar Alshibely, Albara A Dabroom, Hussam M Algarni, Mohammed S Alshehri

**Affiliations:** 1 Orthopaedic Department, King Saud Bin Abdulaziz University for Health Sciences, Jeddah, SAU; 2 Orthopaedic Surgery, King Abdulaziz Medical City, Ministry of National Guard - Health Affairs, Jeddah, SAU

**Keywords:** anterior cruciate ligament (acl), acl, anterior cruciate ligament (acl) reconstruction, return to sport, sports participation

## Abstract

Background

Physically active individuals are susceptible to sports injuries, one of which is anterior cruciate ligament (ACL) injury. ACL injury can be managed conservatively or by surgical reconstruction. Returning to sport (RTS) after ACL injury is one of the main goals of ACL reconstruction (ACLR). However, rates of return vary and can be affected by several factors. The objectives of this study were to estimate the rate of return and to identify the factors that might affect RTS after ACLR.

Methods

This was a cross-sectional study, including individuals who had an ACLR. Participants were sent an online survey included questions about their injury, sport participation, International Knee Documentation Committee form (IKDC), and the Tampa Scale for Kinesiophobia (TSK-11). Participants who had their surgery in the period between January 2011 to December 2018 and participated in sports regularly were included. Descriptive statistics were performed. Chi-square and student t-tests were performed to explore the differences between participants who returned and the ones that did not.

Results

A total of 93 participants were included. The majority (69.9%) were playing soccer before the injury. Though more than half (61.3%) returned to sports, only 29% participated at the same level before the injury. Fear of reinjury was the most frequent reason for delaying or not returning (30%), followed by pain (29). Significantly better IKDC (p=0.002) and TSK-11 (p<0.001) scores were noted in participants who had returned to sports. On the other hand, participants’ age, body mass index (BMI), time from injury to surgery, time since surgery, and times of sports participation per week were not found to be significantly different between those who returned versus those who did not.

Conclusion

The participants in this study had a low rate of return with fear of reinjury being the most common reason not to return. However, a participant’s IKDC and TSK-11 scores were associated factors for RTS, thus optimizing those factors after surgery is crucial.

## Introduction

The anterior cruciate ligament (ACL) and posterior cruciate ligament (PCL) of the knee maintain the rotational and anteroposterior stability of the knee joint [1]. An ACL injury frequently occurs, through contact and non-contact mechanisms [2,3]. The most prevalent non-contact movements associated with ACL tears, are abrupt pivoting, twisting, and cutting movements during intense sports [3,4]. The management of an ACL tear may be surgical or non-surgical [5]. Management depends on several factors such as the activity level, occupational demand, and adherence to a rehabilitation protocol [6]. Non-surgical management is reserved for individuals with a sedentary lifestyle and minimal risk of re-injury [6]. Surgical treatment is reserved for active individuals with a higher risk of re-injury and the optimal management strategy requires careful consideration of all the factors [6]. To this end, ACL reconstruction (ACLR) surgery is considered the gold standard modality for active patients [5]. During the ACLR surgery, the torn ACL is replaced with an autograft or allograft tissue [4]. Several factors may affect the outcome of ACLR surgery such as the timing of ACLR surgery, graft selection as well as pre- and post-operative rehabilitation [4]. Early ACLR increases the incidence of developing joint stiffness, however, delayed ACLR decreases muscle strength and increases the incidence of additional injuries [7,8]. Current evidence indicates that the ACLR should be performed within five months of post-ACL injury [9]. Non-surgical management consists of rehabilitation, functional knee bracing, and activity modification [6]. Irrespective of the management, the implementation of a pre- and post-operative rehabilitation program is crucial for the success of ACLR management [6].

Evans and Nielson reported that for patients who had ACLR surgery, the return to sport (RTS) was variable [3]. The average time to resume activity is six to twelve months after the ACLR surgery [8]. However, the nature of the post-injury activity and the patient’s rehabilitation progress may affect the average time to return to activity [8]. According to Ardern et al., 90% of patients had a successful outcome after ACLR surgery [10]. However, irrespective of the high rate of successful surgical outcomes and physical improvement with rehabilitation, only 63% of patients returned to their pre-injury level of activity [4,11]. The discrepancy between successful surgical outcomes and return to pre-injury level of activity may be due to psychological factors [10].

RTS after ACLR surgery depends on physical, choice-related, and life-related factors [12]. The most frequent factor for not returning back to sports after the ACLR surgery was persistent knee symptoms, specifically pain, and swelling. Other less frequent persistent knee symptoms include stiffness, instability, and weakness [12]. A choice-related factor like kinesiophobia or fear of re-injury is also a frequent cause [12]. Fear of re-injury was correlated with RTS post-ACLR surgery. Patients with a high level of fear of injury are less likely to RTS and the group with low levels of fear of injury are more likely to RTS post-ACLR surgery [13]. Less frequent choice-related reasons were the belief of being advised not to return to their previous activities, lack of interest, and lack of time [12]. Social-related factors include factors such as comorbidities, as well as educational and vocational demands [12]. In addition, RTS is associated with age and gender, as younger athletes with an ACL tear, were more likely to RTS [14]. To expand the current understanding of factors associated with RTS post-ACLR surgery, additional research is justified [10].

This study aimed to evaluate the RTS participation in patients who had ACLR surgery. To our knowledge, this is the first study to assess RTS in a Saudi population.

## Materials and methods

Study design and participants 

A retrospective review with a cross-sectional design was conducted in an orthopedic department of a tertiary hospital. The inclusion criteria included ACLR surgery and participating in sport regularly before the injury. The hospital’s electronic records from January 2011 to December 2018 were reviewed. There were 553 ACLR surgeries performed in this period. Prospective participants were contacted with a mobile message containing an invitation to participate in the research and a link to an online survey to be completed. Reminders were sent after three days and a week later.

Study measures

Demographic Information and Injury History

Participants’ age at the time of doing the online survey, gender, marital, and employment status were collected. Information related to the injury and surgery included the side of the injury, time elapsed between injury and surgery, number of clinic visits before the surgery, time since surgery, and a concomitant knee injury.

Sport Participation

The variables included the frequency of sport participation per week, type of sport participation before the injury, and returning or not to sport. For the group who returned to sport, questions were posed relating to whether the level of sport participation is the same as before the injury and the length of time before resuming sport. Lastly, the reasons for delaying or not returning back to sport were explored. 

Subjective Knee Function

A part of the online survey was an Arabic validated version of the International Knee Documentation Committee’s (IKDC) subjective knee evaluation form [15]. The IKDC was validated for an assessment of knee function after an ACLR [16]. The score ranges from 0-100 with a higher score indicating better knee function.

Assessment of Kinesiophobia

The last part of the online survey included an Arabic validated shortened version of the Tampa Scale for Kinesiophobia (TSK-11) [17]. TSK-11 was designed to assess pain-related fear of movement; however, it has been proven to be a valid and reliable instrument for the assessment of kinesiophobia and fear of reinjury after ACLR [18]. The shortened TSK-11 version contains 11 items with the lowest score of 11 which indicates negligible or no kinesiophobia and fear of reinjury and the highest score of 44 which indicates extreme kinesiophobia and fear of reinjury [18].

Statistical methods and ethical consecrations

Statistical analyses were conducted with IBM Statistical Software for Social Sciences (SPSS) for Windows, version 25 (IBM Corp., Armonk, NY, USA). Descriptive statistics were performed. RTS was considered a dependent variable. Continuous variables (age, body mass index (BMI), time from injury to surgery, time since surgery, times of sport participation, and IKDC and TSK-11 scores) were presented as mean and standard deviation and compared using independent sample t-test. Categorical variables (marital and employment status, side of the injury, concomitant knee injury and type of sport) were presented as frequencies and percentages and associations between them and RTS were assessed using Chi-square test for independence. A p-value of less than 0.05 was considered significant. The study was approved by the Institutional Review Board of King Abdullah International Medical Research Center. Participants’ completion and submission of the online survey was considered as informed consent.

## Results

Demographic information and sample characteristics

A total of 553 participants were contacted and 117 of them responded (response rate, 21%). Out of the responders, 93 participants were included, with the majority being male (98.9%). The mean age was 35.24 ± 6.74 years with mean BMI 27.91 ± 4.52 kg/m2. The majority (78.5%) were married and 90.3% employed. More than half (51.6%) injured their left knee. The majority (81.7%) did not have any concomitant knee injury. The mean time from injury to surgery was 22.6 ± 32.2 months and, at the time of the study, the mean time since the surgery was 4.47 ± 2 years. The mean number of hospital visits after the injury but before the surgery was 4.84 ± 4.64 visits.

Sport participation and return

Soccer was participated in most frequently before the injury (69.9%), followed by jogging and running (16.1%), volleyball (6.5%), weightlifting (1.1%), and (6.5%) other sports. The frequency of sport participation per week was 4.43 ± 2.2. More than half (61.3%) returned to sport after the surgery but the level of sport was, for just 29.1%, the same as before the injury. The most frequent reasons for delaying or not returning to sports were fear of reinjury (30.1%), followed by pain (29%) and lack of interest (21.5%); more reasons are presented in Table 1

**Table 1:** Reasons for Delaying or not Returning to Sport

Reason	n (%)
Fear of reinjury	28 (30.1)
Pain in the joint	27 (29)
Lack of interest	20 (21.5)
Joint instability	19 (20.4)
Employment or education	17 (18.3)
Not enough time	17 (18.3)
Joint stiffness	15 (16.1)
Joint swelling	14 (15.1)
No activities available	11 (11.8)
Family and social reasons	9 (9.7)
Doctor advised me not to return	6 (6.5)

 At the time of the survey, the mean IKDC score was 60.7 ± 17.9. The mean TSK-11 score was 27.6 ± 8.2. 

Factors associated with RTS

When using independent sample t-test, there were no significant differences in age, BMI, time from injury to surgery, time since surgery, and times of sport participation per week between participants who had returned to sport and those who had not. However, participants who had returned had significantly higher IKDC score (p = 0.002) and lower TSK-11 score (p < 0.001) than participants who had not, indicating better knee function and low fear of reinjury to be strongly associated with RTS. Moreover, no significant associations were found between RTS and marital or employment status, side of the injury, or concomitant knee injury when using the Chi-square test. More details in the comparison between the two groups are demonstrated in Table 2. 

**Table 2:** Comparison Between Participants Who Had and Had Not Returned to Sport *P < .05, **P < .01, SD: standard deviation, y: year, kg/m: kilogram/meter, m: month, BMI: body mass index, IKDC: International Knee Documentation Committee, TSK: Tampa Scale for Kinesiophobia.

	Returned to Sport, Mean (SD) or n		
	Yes (n = 57)	No (n = 36)	P Value
Age, y	35.6 (7.5)	34.6 (5.3)	.46
Married: not married	44:13	29:7	.7
Employed: not employed	53:4	31:5	.27
BMI, kg/m^2^	27.7 (4.5)	28.2 (4.5)	.6
Right knee: left knee	29:28	16:20	.54
Time from injury to surgery, m	19 (29.3)	28.7 (36.2)	.18
Time since surgery, y	4.3 (2)	4.7 (1.9)	.35
Concomitant knee injury, yes: no	11:45	4:32	.28
Times of sport participation	4.2 (2.1)	4.7 (2.2)	.34
IKDC score	65 (16.6)	53.6 (17.9)	.002^**^
TSK-11 score	25.1 (8.2)	31.6 (6.4)	.001^**^

## Discussion

The sample was predominantly male (98.9%) with a mean age of 35.24 years, supporting literature which reports that ACL injuries most frequently affect young males. In Saudi Arabia, mostly males participate in sports, clarifying the gender gap within the cultural conventions of the country [19,20].

The primary outcome of the current study was the 61.3% rate of RTS, marginaly lower than studies reporting rates 66% to 67% and substantially lower than 97% reported in another study [21-23]. A possible justification for the lower RTS rate in our study is that the majority of our sample are not professional athletes, as professional athletes usually return to sport in higher proportions [24]. More than half of the current sample (69.2%) played soccer before being injured, and soccer is known to have a lower RTS rate, compared to bicycling or jogging [25]. Roos et al. reported that soccer has a low RTS as only 30% still play soccer after three years and only 20% after seven years [26]. However, there is not enough evidence to accept that the RTS is sport dependent, as it was reported that males had a higher rate of RTS, which is not supported by the current results [10,26].

In terms of the rate of returning to the pre-injury level of sport, the current study found that 29.1% of the group who returned to sport, participated at the pre-injury level. The rate is lower than Ardern et al. (41%) or Webster et al. (61%) [22,27]. The subjectivity of “same pre-injury level” might be a contributing factor to the varying levels reported as well as recall bias. In the current study, the fear of reinjury was the primary cause for not returning to sports (30.1%). Similarly, other studies also report that the fear of reinjury plays a major psychological role and is the most frequent reason for not returning to sport participation [12,13].

The current study reported the time between the injury and the surgery to be 22.6 months, with a prior systematic review reporting periods ranging from 7.8 months to 35 months [21]. The IKDC score of our study was 60.7, in contrast to a study performed in Australia with 122 athletes, reporting a mean IKDC score of 85 [27]. In this study, number of times of sport participation per week was not a significant associated factor for RTS. Similarly, Ardern et al. found no significant correlation between the level of sport pre-injury and return [27]. Participants who had returned to sport had significantly better IKDC and TSK-11 scores than those who had not returned, which indicates better knee function and less fear of reinjury and kinesiophobia. These findings were supported by Webster et al. and Kvist et al. studies [13,28]. Similar to our results, the patient’s age was not found to be correlated with return in three different studies [4,28,29]. Kvist and colleagues reported a significantly higher rate of return in individuals who had ACLR with six months of ACL injury [13]. On the other hand, no significant difference in return was related to time from injury to surgery. Concomitant knee injury was not correlated with RTS in this, and a similar study [28].

The results of this study and previous studies highlight the multifactorial nature of resuming sports after ACLR.

The findings of the study should be considered in the context of some limitations. The study used a self-administered questionnaire completed on an online platform. The study has a limited sample size due to the low response rate of the participants. Finally, the study lacks female representation which limits the applicability to societies with higher sport participation in females. Based on the findings, we recommend addressing psychological concerns in the period after surgery to encourage RTS participation.

## Conclusions

The study demonstrated a low rate of RTS after ACLR, with a follow-up period of one-to-eight years. Psychological aspects played a significant role; fear of reinjury was the most frequent cause of not returning to sport. Significantly better IKDC and TSK-11 scores were discovered in participants who had returned to sport. RTS after ACLR is affected by multiple factors, requiring additional research.
